# Challenges and Opportunities in the Implementation of the Family Adoption Program Under the Competency-Based Medical Education in India: A Qualitative Study

**DOI:** 10.7759/cureus.102748

**Published:** 2026-01-31

**Authors:** Dhananjay Kumar, Himanshu K Bhaiya, Surendra Sahu

**Affiliations:** 1 Department of Community Medicine, Sheikh Bhikhari Medical College, Hazaribag, IND; 2 Department of Medicine, Sheikh Bhikhari Medical College, Hazaribag, IND; 3 Department of Community Medicine, ESIC Medical College and Hospital, Ranchi, IND

**Keywords:** descriptive qualitative study, family adoption, medical education curriculum, rural health, teaching competencies

## Abstract

Background and objectives: The Family Adoption Program (FAP) is an initiative under the competency-based medical education (CBME) in which each medical student is assigned to families in a rural community from the beginning of the curriculum. Community engagement in medical education gives the students an insight into the living conditions of the public and how they influence their health, along with improving their communication skills. This program provides preventive and primary care services to resource limited rural population. The objective of this study was to recognize perceived challenges and benefits of the FAP to the medical students and to the adopted families, and to gather suggestions for the improvement of the FAP.

Methods: This observational qualitative study was conducted using a purposive sampling technique. Faculty members, medical social workers (MSWs) of the community medicine department, along with first, second, and third year medical undergraduate students, and heads of allotted families were our study subjects. Key informant interviews (KIIs) and the focus group discussion (FGD) technique were the study tools. A total of 38 KIIs (18 faculty members, 5 MSWs/field staff, and 15 community members) and three FGD sessions were conducted.

Results: The participants felt that the FAP provides a good opportunity for rural people to improve their health through health education activities and health camps. The undergraduate students realize that the FAP will have a good and positive impact on medical education. The most important challenge expressed by participants was the shortage of human resources.

Conclusions: The FAP has a good scope to improve rural health due to long-lasting bonding between students and rural people and health-promoting activities. The FAP improves communication skills and develops empathy among students. There is a need to strengthen these activities through improving infrastructure and increasing manpower.

## Introduction

In India, around 65% of the population resides in rural settings, whereas the availability of healthcare facilities is skewed toward urban setups. Hence, there is a need to take measures to make healthcare more accessible to rural and needy populations and to impart community-based, community-oriented training to budding healthcare professionals [1,2]. The Family Adoption Program (FAP) is a new concept, which is being implemented under the competency-based medical education (CBME) for medical undergraduate students in India, implemented by the National Medical Commission (NMC), the regulatory body for medical education in India. Families are being allotted to the students in rural areas from the beginning of their Bachelor of Medicine, Bachelor of Surgery (MBBS) curriculum [3]. The FAP is recommended as a part of the curriculum of community medicine and begins from the first professional year and remains throughout the curriculum.

Community engagement gives medical students a firsthand experience of the living conditions of the people they encounter as patients in the hospital. The students also understand how various determinants of health influence patients in real life [3]. One of the goals of the undergraduate medical education is to make the services of medical professionals accessible to all citizens across geographical boundaries. Considering this, every medical college allots a minimum of three households in a village to all the first-year MBBS students under the FAP. Students regularly visit their allotted families up to the third year of their undergraduate curriculum to be groomed as "complete doctors" with qualities such as confidence, empathy, and leadership in sociohealth fronts [4].

The FAP is a new introduction in the CBME. Like every new program, this also needs evaluation at regular intervals. There are also gaps in existing literature about various advantages and loopholes in this program. This study tried to find different challenges and opportunities during the implementation of this program.

This program also gives some other opportunities to find new scope to improve the learning process in a community setting. So, this study can help in finding these challenges to make suitable changes to make this program successful for students as well as community members. We can be in a better position to implement this program effectively if we find challenges and their possible solutions in the upcoming years. The new opportunities can be utilized in further implementing the curriculum for medical graduates.

Research question

What are the different challenges and benefits of the FAP to medical students and to the adopted families?

Objectives

This study aims to recognize perceived challenges and benefits of the FAP to the medical students and to the adopted families, and to gather suggestions for the improvement of the FAP.

## Materials and methods

Study design and setting

This was an observational qualitative study conducted from February 2024 to June 2024 at Sheikh Bhikhari Medical College (SBMC), Hazaribag, after clearance from the institutional ethics committee of SBMC Hazaribag vide letter no IEC/13/2024 dated 06/03/2024. SBMC Hazaribag is a relatively new medical college established in 2019. This is a government medical college situated in the northern part of Jharkhand, India. Purposive sampling was used to accomplish the study.

Study participants and sample size

There are three major stakeholders of the FAP: undergraduate students, allotted family members, and faculty and field staff. MBBS students of first, second, and third year (i.e., Phase I, II, and Phase III Part 1), MSWs of SBMC, Hazaribag, were the study participants. Faculty members of the community medicine department of different medical colleges (both government and private) were also our study participants. In-depth interviews of faculty members and heads of families of different allotted families were conducted until data saturation was achieved. We have taken an interview of 18 faculty members selected randomly from 18 different medical colleges of Bihar, Jharkhand, Uttar Pradesh, and West Bengal states of India, who were the nodal person of FAP at their colleges. A total of 18 faculty members, five MSWs/field staff, 15 heads of the families of different allotted families, and 26 undergraduate students participated in this study, who were selected randomly.

Study tool and sampling procedure

Inputs were taken through key informant interviews (KIIs) for faculty and MSWs, while focused group discussions (FGDs) were conducted with MBBS students. The KII of faculty members of different medical colleges was done telephonically, while the KIIs with MSWs were conducted in person. The KII with the head of the allotted families was also done in person. The KIIs/FGDs were recorded throughout. Currently, three batches are undergoing the FAP. A total of three FGDs were conducted, i.e., one from each professional year. Only willing students, given their consent for participation, were the participant of the study. The participants of FGD ranged from eight to 10. The duration of KII and FGDs ranged from 30 to 40 minutes. A total of 38 KIIs (18 faculty members, 5 MSWs/field staff, and 15 community members) and three FGD sessions were conducted.

The participants' consent for recording was verbally obtained before the recording. They were also informed of their right to drop out of the KII/FGD at any point. The KIIs/FGDs were conducted based on the basis of a guide in which provision and scope for newer inputs were kept and probed. The interview questionnaire was developed by the research team with the help of the head of the department of community medicine and some other departments of SBMC, Hazaribag. The questionnaire was tested and validated through pilot testing on some study participants.

The FGD/KII guide was prepared based on the following themes: (a) experiences so far during the FAP, (b) different advantages of the FAP as per participants, (c) different challenges of the FAP as per participants, and (d) different measures to improve the FAP.

Data analysis

As this was a qualitative study, data were collected through an interview technique and an FGD, and it was recorded. The recordings of KIIs and FGDs were transcribed properly. All the lines and parts of lines contributing to the research question were marked as codes. The codes were then categorized under various themes. The coding and thematic analysis were done manually. During analysis, these recorded data were analyzed based on selected themes. This exercise was done rigorously so that any important finding could not be left out. Some of the verbatim quotes of the participants are presented as quotable quotes.

## Results

A total of 18 faculty members, five MSWs/field staff, 15 community members, and 26 undergraduate students participated in this study. A total of 38 KIIs and three FGDs were conducted. The FGDs and KIIs were based on the following themes: (1) overall experience of the FAP, (2) advantages of the FAP, (3) challenges of the FAP, and (4) measures to improve the FAP.

Theme 1: overall experience of the FAP

All the participants reported a good experience of the FAP, and the inclusion of the FAP in CBME is a welcome step. For the students, it was a new learning opportunity. A social worker said, “Initially, in a new locality, everyone asked about our purpose, the advantages they would get, but now everyone knows us and supports us in FAP.” A villager said, "It’s a new experience for us. Medical students and health professionals are coming to our houses…we cannot imagine. We are very happy."

Theme 2: advantages of the FAP

The participants felt that the FAP would have a good and positive impact on medical education in various ways. Almost all faculty and field staff accepted that the FAP provides a good opportunity to rural people in improving their health through health education activities and health camps. Health camp also provides an opportunity for screening of various noncommunicable diseases like diabetes and hypertension. As told by one faculty, “The family adoption program is useful to rural people because they will get health education regularly.” A MSW said that “Frequent health camps will help in improving people’s health.” Another MSW said that “The students will help rural people to avail health facilities in medical colleges.” One faculty member said, “This program is quite useful to students of affluent classes, as they have no idea about patients’ backgrounds.” This program also improves the communication skills of medical undergraduates. As told by one faculty, “they are learning how to talk with people, especially the underprivileged class.”

During the FGD, students also accepted that the FAP is providing the opportunity to learn about the rural environment and their health in a better way. This is also motivating them to do something for the family and the country. As told by one student and agreed by others, “How can we do our best to help them?” Another said, “The spirit of doing something for the people and the country motivates us.” In another FGD, a student said, “A lot of misconceptions about health among the general public. How much they are relevant…how they affect health…we see now.” Another student told about the FAP advantage, like “Hospital communication is in a different environment… something like unreal. Only the most important things are spoken. Here, the family can talk freely with us.’’ A student said, “We now see important health problems in the real world rather than studying them in textbooks.”

The interview with villagers pointed out that this program is very beneficial to them. They are utilizing the health facilities through regular medical camp conduct. They are also getting benefits during treatment at medical colleges due to contact with medical students. As one of the villagers told, "they come and tell us about healthy habits, what to eat, and preventive measures. We are learning from them." Another one said, "We have benefited from the camp. My blood pressure and blood sugar were checked for the first time. This program should continue." Another one said, "My child was very sick, then I called the student who came to our house. He arranged a bed for my child in the medical college. I am grateful to him."

Theme 3: limitations/challenges of the FAP

The most important challenge expressed by participants was the shortage of human resources. As one faculty said, “In the scarcity of faculties, we are already burdened by SDL and SGDs, and now this FAP, we need more faculties and staff.” Another said, “This is a good initiative, but we need more faculty, staff, and MSWs to engage the students.” An MSW said, “To watch so many students in the field, we need more supporting staff.” Other logistical issues were also explored regarding the implementation of the program. An MSW said, “More instruments like a BP machine, a weighing machine, etc., are required during visits to the selected families because they asked for facilities.” Another one said, “For weighing weight and blood pressure measurements of family members, more equipment is required.”

The safety of students in the community is another issue raised by faculty. Unavailability of family members was also an issue as one participant of the FGD told that “on several visits, I was unable to find 3-4 members of the family because they were out for their job at that time.” An MSW also told that “after allotment of family, on subsequent visit we found a few houses closed. This leads to a change of family to that particular student.”

Some people were hesitant to disclose their total income. This leads to assess their socioeconomic status difficult. During the FGD, one student said that “family members are quite hesitant to tell their family income because they think that this will lead to cessation of their various government schemes.”

Acceptance of students by the families was not a big issue in most families, but a few students reported about the caste and religion factor during the FGD. As told by one student, “Initially, they were supportive, but after knowing my religion, they became hesitant.” Another one told “Yes, sir, they asked my caste and after knowing that we are from the same caste, they offered me juice also.”

We asked about the challenges/problems faced by villagers in the FAP. The major concern from villagers was about the questions asked by students related to socioeconomic status, mostly during the initial part of their visit. Language barrier was also an issue in a few cases. As one of villager told, "on the very first day, they asked about my profession and my income. I was unable to understand why this was asked."

Theme 4: measures to make FAP more effective

The participants suggested several measures that can be taken to make the FAP more effective. Almost everyone said that health camps should be organized in selected villages on a regular basis. Health awareness activities should also be done. As told by a faculty member, “Planning and arrangement of health camps should be on a regular basis.” An MSW said, “A health camp will generate belief among rural people; this will make the FAP more effective.” Another MSW said, “More health awareness activities like roleplay and nukkad natak should be done.” Some faculty advised that the orientation of students and staff should be done before going to the village. This would clarify the role of students and would also be helpful in lowering people's expectations. A faculty member said, “Orientation is needed for a few sessions regarding how to engage with them.” Another faculty member said, “Students and staff should clarify that they are first-year students, not doctors. It will lower the expectations of the population.” It was also advised that the evaluation of the FAP should be done. “Proper evaluation and grading should be done; otherwise, many students will not take it seriously.” It was also suggested that if a patient from an adopted village goes to the hospital, they must be seen on a priority basis. A faculty member suggested, “Tertiary care follow-up in the hospital is a must. Otherwise, they will lose trust in no time.”

We asked what other measures should be taken to make this FAP more effective. Everyone said that the health camp should continue, and it should be more frequent. They also said that medicines for different ailments should be available to them. They also said that villagers must be given preference in case of a hospital visit. One of the villagers said, "The health camp is a good initiative. More frequent health camps should be organized." Another said, "We have to face a long queue in the hospital. We must be given priority in that." Major findings are summarized in Table 1.

**Table 1:** Major findings of the thematic analysis of the qualitative data FAP: Family Adoption Program; KII: key informant interview; FGD: focused group discussion

Themes	Major findings of KII conducted with faculties and medical social workers	Major findings of KII conducted with villagers	Major findings of FGDs conducted with undergraduate students
Theme 1: Overall experience of FAP	Good experience with the Family Adoption Program	New and welcome step	New learning curve for students
Theme 2: Advantages of the Family Adoption Program	1. Benefit to rural people through health education and health camps. 2. Screening of various noncommunicable diseases like diabetes and hypertension	1. Benefit during the health camp. 2. Health education from students	1. Learning healthcare issues in a rural setting. 2. Understanding the role of health in family and community. 3. Motivation to do something for the family
Theme 3: Limitations/challenges of FAP	1. Shortage of human resources. 2. Shortage of logistics, like field instruments and medicines. 3. Student safety issue	1. Language barrier. 2. Sometimes asked more personal questions	1. Hesitation to share income data by families. 2. Locked houses during revisits. 3. Caste and religion discrimination
Theme 4: Measures to make FAP more effective	1. Health camps should be organized on a regular basis. 2. More manpower and logistics should be provided	1. More health camps are required. 2. Availability of more medicines. 3. Preference during hospital visit	1. Health awareness activities should be done. 2. Preference of family members in hospital

## Discussion

The present study tried to find various advantages, challenges, and opportunities in the implementation of the FAP under the NMC's new CBME. Patient satisfaction in rural health training centers was limited [5]. This program, i.e., the FAP, may improve satisfaction.

Overall, students have a good exposure to the rural environment during the FAP. Most of the students from an urban background and well-to-do families came in contact with rural people, with most of them coming from poor socioeconomic backgrounds. This was a totally new experience for them. Students as well as community members are both benefited by the FAP. Students came to know about the rural environment and health conditions in relation to social and environmental factors. They are also developing communication skills. By participating in different screening programs and generating awareness about national health programs, medical students will learn about the Indian healthcare delivery system at the grassroots level. Community participation is a key factor that enables effective health system functioning and is the first step toward true community empowerment in health [6]. Community settings provide a comprehensive learning experience to undergraduate students, which is somewhat lacking in hospital-based teaching [7].

On the other hand, rural people are getting health awareness. They are also utilizing the services of health camps. They have better access to a medical college with support from medical students. Community will also have greater access to health care, improved health care-seeking behavior, and positive health, which will allow us to ultimately reach our goals of Health for All [2].

Exposure to diverse familial backgrounds improves the medical student’s cultural sensitivity, thereby reducing disparities in healthcare and fostering a culturally competent approach to patient management. The expectations of faculty and support staff addressed in this study were similar to those identified in other studies [2,8-10]. The safety of students is also an issue. As students are going to families on a large scale, it’s been difficult for field staff and faculty to monitor every student. So, there is always a safety issue involved in the FAP.

The FAP also aims to improve students’ communication skills, cultivate empathy toward adopted families and rural populations, develop leadership in healthcare provision, instill responsibility as primary consultants, and teach basic clinical skills [11-13]. To improve FAP, more health camps and health awareness activities should be conducted in rural areas. Students and faculty, along with field staff, suggested more frequent visits in allotted families. Adequate human resources are required for student support and guidance, thereby maximizing the program’s learning impact [14]. Similar concerns were reported by Yalamanchili et al. [2], Landge et al. [12], Shah et al. [15], and Sulgodu Ramachandra et al. [16].

Limitations of the study

Due to time and budgetary constraints, only one college is included for the FGD in the study. The inclusion of more medical colleges might have provided a clearer picture of the different aspects of the FAP. A face-to-face interview could be better in comparison to a telephonic interview with faculty members of other institutes. Social desirability bias might also affect this study during the FGD and in-depth interview; however, all steps were taken to minimize this bias. These may be considered as a limitation of this study.

## Conclusions

The FAP provides benefits to both MBBS students and community members. It improves communication skills and develops empathy among them. Community members benefited through health education and health check-ups. There is a need to strengthen these activities through improving infrastructure and increasing manpower. Regular health camps are required, and the adopted family should be prioritized during hospital consultation.

## Appendices

Themes/questions for the key informant interview and focus group discussion

1.     Name:

2.     Age:                                                                   

3.     Place:

4.     Current position:

5.     As the Family Adoption Program is a new initiative, how is your experience related to this?

6.    According to you, what are the possible benefits of this family adoption program?

7.   What are the challenges you are facing during the implementation of this program?

8.   What are possible solutions to these problems?

9.   What can be done to make this program more effective?

10.  According to you, what are the other possible scopes of this program?
